# Detection and genotyping of *Trypanosoma cruzi* from açai products commercialized in Rio de Janeiro and Pará, Brazil

**DOI:** 10.1186/s13071-018-2699-6

**Published:** 2018-04-10

**Authors:** Renata Trotta Barroso Ferreira, Maria Luiza Cabral, Ronald Sodré Martins, Paula Finamore Araujo, Sérgio Alves da Silva, Constança Britto, Maria Regina Branquinho, Paola Cardarelli-Leite, Otacilio C. Moreira

**Affiliations:** 1 Instituto Nacional de Controle de Qualidade em Saúde, Fundação Oswaldo Cruz, Rio de Janeiro, Brazil; 2 Laboratório de Biologia Molecular e Doenças Endêmicas, Instituto Oswaldo Cruz, Fundação Oswaldo Cruz, Rio de Janeiro, Brazil

**Keywords:** Chagas disease, *Trypanosoma cruzi*, Açai, Oral transmission, *T. cruzi* genotyping, Health surveillance

## Abstract

**Background:**

Several cases of food-borne acute Chagas disease (ACD) have been reported in the Brazilian Amazon so far. Up to 2004, the occurrence of ACD by oral transmission, associated with food consumption, was rare. Recent cases of ACD in Brazil have been attributed to the consumption of juice from the açai palm containing reservoir animals or insect vectors waste, infected with *Trypanosoma cruzi*. This study aimed to determine the *T. cruzi* contamination rate and to genotype the parasite in food samples prepared from açai, which are commercialized in Rio de Janeiro and the Pará States in Brazil.

**Methods:**

The amplificability of DNA extracted from açai samples, and *T. cruzi* and Triatominae detection were performed by conventional PCR. Molecular characterization was done by multilocus PCR analysis, to determine the parasite discrete type units (DTUs) based on the size of PCR products in agarose gels, using the intergenic region of the spliced leader (SL), 24 Sα rDNA and nuclear fragment A10 as targets.

**Results:**

From the 140 samples of açai-based products analyzed, *T. cruzi* DNA was detected in 14 samples (10%); triatomine DNA was detected in one of these 14 samples. The parasite genotyping demonstrated that food samples containing açai showed a mixture of *T. cruzi* DTUs with TcIII, TcV and TcI prevailing.

**Conclusions:**

In this study, the molecular detection and identification of *T. cruzi* from açai-based manufactured food samples, was performed for the first time. Although parasite DNA is a marker of possible contamination during food manufacturing, our findings do not indicate that açai is a source of Chagas disease via oral transmission per se, as live parasites were not investigated. Nevertheless, a molecular approach could be a powerful tool in the epidemiological investigation of outbreaks, supporting previous evidence that açai-based food can be contaminated with *T. cruzi*. Furthermore, both food quality control and assessment of good manufacturing practices involving açai-based products can be improved, assuring the safety of açai products.

## Background

Chagas disease (ChD) is an important neglected tropical illness caused by the flagellate protozoan *Trypanosoma cruzi* (Kinetoplastida: Trypanosomatidae). The disease is established through a complex biological cycle, including insects belonging to the subfamily Triatominae (Hemiptera: Reduviidae) and mammalian reservoir that may belong to several classes, such as marsupials and rodents [1]. Currently, more than five million people are infected and approximately 70 million living at risk in the world [2]. In the chronic phase, the disease shows a diversity in clinical manifestations, from indeterminate to cardiac and/or digestive forms [2, 3], which can be associated with complex interactions between the genetic diversity of the parasite and the host, and environmental and epidemiologic factors [4].

The reemergence of ChD from 2005 challenged Brazilian authorities. This disease used to be characteristic of rural areas, especially the most deprived populations without access to adequate sanitary conditions; however, in recent years, it has also been disseminated in urban areas, with transmission via ingestion also reported. In this case, the disease is more aggressive and difficult to control, with lethality as high as 5% in the Amazon [5]. In Brazil, several cases of acute Chagas disease (ACD) have been reported as outbreaks, characterized by groups of individuals gathered in the same place, who ingested the same food and became sick almost simultaneously, with fever and general manifestations of a systemic infection [6]. Despite the increasing number of acute cases, reports of this form of the disease are rare in the literature.

The açai palm is most commonly associated with ACD cases in the northern Amazon and involves either contamination of the fruits or the pulp itself, via reservoir animal waste or infected insect vectors in endemic areas [7, 8]. Consequently, the ability to produce sanitary and high-quality food stuffs from the açai palm concerned the authorities and created the need for public health strategies to combat Chagas’ disease and its spread via this novel route. In cases of ACD outbreaks, elucidation of oral transmission is usually determined via clinical and/or epidemiological investigations. However, there were no official methods established for this kind of investigation in Brazil.

Molecular methods based on PCR can be used to test food samples for pathogens, or residual pathogen DNA. This is usually the case following outbreaks, due to the delay between the event and analysis. Thus, the availability of methods for the detection of *T. cruzi* in food samples would be a powerful tool in the epidemiological investigation of Chagas disease.

## Methods

### Samples

One hundred and forty samples of açai-based products were analyzed in this study, in two different periods. In the first period (2010), 47 samples were analyzed, including 17 samples gathered randomly from food markets in Rio de Janeiro, 9 samples of açai juice, 8 frozen or chilled samples of açai with guaraná or fruits, and 30 samples gathered from street markets in Pará. Those included 2 samples of açai pulps, 9 açai juices, 1 açai candy, 1 chocolate bonbon with açai, 1 açai ice cream, 1 açai popsicle, 2 açai with rice porridge, 2 açai seeds, and 11 açai fruits. During the second period (2011–2015), 93 samples were gathered from Rio de Janeiro and Pará food markets by local regulatory health authorities. In Rio de Janeiro, 48 samples consisting of 27 açai juices, 1 açai pulp, and 20 açai with guaraná or fruits were collected during 2011 and 2012. In Pará, from 2012 to 2015, 20 açai pulps, 4 açai juices and 20 açai fruits were collected, as part of a monitoring program to evaluate the sanitary conditions of street markets. In 2013, a sample of açai pulp was personally collected and sent to the laboratory.

For the purposes of sensitivity and specificity assays we obtained samples of *Trypanosoma*, *Leishmania*, bacteria, yeast, and fungi. Specifically, *Trypanosoma cruzi* (strains or clones Y, Dm28c, CL Brener, INPA 222, INPA 4167, COLTRYP 016, COLTRYP 043, COLTRYP 370), *T. rangeli* (COLPROT 273), *T. cervi*, *T. lewisi* and *T. mega* were supplied by the Coleção de Protozoários (COLPROT) and by the Coleção de Trypanosoma de mamíferos silvestres, doméstico e vetores (COLTRYP), which belong to the Oswaldo Cruz Institute (Rio de Janeiro, Brazil). We obtained *Leishmania amazonensis* (IOC-L575), *L. braziliensis* (IOC-L560), *L. guyanensis* (IOC-L565), *L. lainsoni* (IOC-L1023), *L. naiffi* (1365) and *L. shawi* (IOC-L1545) from the Coleção de Leishmania do Instituto Oswaldo Cruz (CLIOC). Finally, the bacteria *Bacillus cereus* (INCQS-00435), *Cronobacter sakazakii* (INCQS-00578), *Escherichia coli* (INCQS-00033), *Staphylococcus aureus* (INCQS-00015), and *Salmonella* sp. (INCQS-00150), the yeasts *Saccharomyces cerevisiae* (INCQS-40001) and *Ogataea polymorpha* (INCQS-400116), and the fungi *Alternaria alternate* (INCQS-40291) and *Botrytis cinerea* (UFPE2802) were provided by the Reference Microorganisms Collection in Sanitary Surveillance (CMRVS) of the National Institute of Health Quality Control (INCQS/ FIOCRUZ). *Trypanosoma rangeli* and *Salmonella* sp. were used as negative controls in the PCR assays.

### Sample preparation and DNA extraction

Açai juice and pulp, açai with guaraná or fruits, açai ice cream, açai popsicles, and açai porridge samples were thawed at 4 °C, homogenized in a Seward Stomacher® 400 Laboratory Blender (Seward, West Sussex, UK), and 2 ml were transferred to a glass vial and frozen at -18 °C for 24 h. Three aliquots were then lyophilized for *c*.18 h. The açai candy sample was manually homogenized, and three portions of 100 mg were transferred to 1.5 ml sterile vials. Before the homogenization of the açai bonbon sample, the chocolate layer was withdrawn, and three 100 mg portions were weighted and moved to 1.5 ml sterile vials. The açai fruits and seeds were placed in plastic bag with 20 ml of sterile water, and the sample was homogenized by inversion 50 times. The water was transferred to a glass vial (100 ml), frozen at -18 °C for 24 h and then lyophilized for *c*.18 h. DNA was extracted in triplicate from homogenized samples using the cetyltrimethylammonium bromide (CTAB) method, as proposed by the Joint Research Center [9], with some modifications [10]. Triplicate sample were analyzed in parallel. The DNA concentrations and quality were estimated by measuring the absorbance at 260 nm (A_260_), 280 nm (A_280_) and 230 nm (A_230_) using the GeneQuant *pro* spectrophotometer (Amersham Biosciences, Buckinghamshire, UK). The average concentration of DNA extracted from açai samples was 70.6 ± 9.8 ng/μl.

### Evaluation of DNA amplificability

The amplificability of DNA (i.e. monitoring for PCR inhibitors) was verified using a plant-specific primer pair VPRBCP1/VPRBCP2 targeting the ribulose 1,5-diphosphate carboxylase/ oxygenase gene (*rbcL*) of the plant chloroplast, as described by Mbongolo Mbella et al. [11]. The reaction was set up in a volume of 25 μl with 1× PCR buffer (pH 8.3), 200 μM of dNTP mix, 1.5 mM MgCl_2_, 0.24 μM of each primer, 1.5 U of Platinum® *Taq* DNA polymerase (Thermo Scientific, Waltham, USA) and 2 μl DNA sample, in a GeneAmp PCR System 2400 thermocycler (Applied Biosystems, Foster City, USA), as follows: 95 °C for 3 min, 40 cycles at 94 °C for 1 min, 60 °C for 1 min, and 72 °C for 1 min with an additional extension at 72 °C for 7 min. Primers were synthesized and purified by Invitrogen (Thermo Scientific). The PCR products were loaded in a 2% (*w*/*v*) agarose gel and stained with GelRed 1× (Biotium, Fremont, USA).

### Detection of *T. cruzi* DNA by conventional PCR

To detect DNA from *T. cruzi* we PCR-amplified the telomeric region of the *gp85*/sialidase superfamily using the primer pair (Tc189F and Tc189R), as described by Chiurillo et al. [12]. The reaction was set up in a volume of 25 μl with 1× PCR buffer (pH 8.4), 0.1% Triton X-100, 160 μM of each dNTP, 1.5 mM MgCl_2_, 0.4 μM of each primer, 1 U of Platinum*® Taq* DNA polymerase, and 2 μl DNA solution, in a GeneAmp PCR System 2400 thermocycler, as follows: 94 °C for 4 min; 33 cycles at 94 °C for 1 min, 60 °C for 30 s; and 72 °C for 40 s with an additional extension at 72 °C for 3 min. The PCR products were loaded in a 2% (*w*/*v*) agarose gel and stained with GelRed 1×.

### Detection of triatomine DNA by conventional PCR

To detect DNA from triatomines we PCR-amplified a portion of the 12S rRNA gene [13]. Conventional PCR was carried out in a final volume of 50 μl, containing: 5 μl DNA solution, 1× PCR buffer, 0.2 mM dNTPs, 4.5 mM MgCl_2_, 1.25 U Platinum® *Taq* DNA polymerase, and 0.1 μM of each primer. Reactions were performed in the GeneAmp PCR System 9700 thermocycler (Applied Biosystems) as follows: 94 °C for 12 min; 36 cycles at 94 °C for 30 s, 55 °C for 30 s; and 72 °C for 30 s with an additional extension at 72 °C for 10 min. The PCR products were loaded in a 2% (*w*/*v*) agarose gel and stained with GelRed 1×.

### *Trypanosoma cruzi* genotyping

**Fig. 1:**
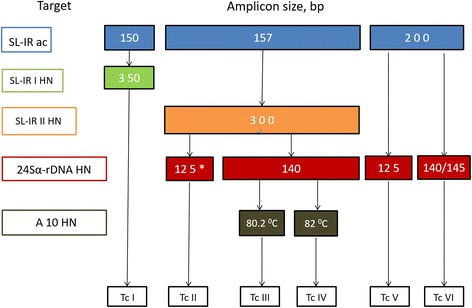
*T. cruzi* genotyping approach by multilocus PCR for DTU assignment. The coloured boxes indicate the expected PCR product sizes (or melting temperatures) for each target, indicated on the left. DTUs (TcI to TcVI) are indicated in white boxes

The molecular characterization of *T. cruzi* DNA extracted from food samples was performed based on multilocus PCR analysis [14] to discriminate between the discrete typing units (DTUs) of the parasite. The identification of genotypes was based on the PCR profile revealed for each target, using the biomarkers spliced leader (SL), 24 Sα rDNA, and nuclear fragment A10 (Fig. 1).

For SL, the intergenic region ac (SL-IR ac) was amplified with primers TCac and UTCC to differentiate *T. cruzi* DTUs TcI (150 bp), TcII, TcV, TcVI (157 bp), and TcIII, TcIV (200 bp). Reactions were performed as follows: 94 °C for 10 min, 3 cycles at 94 °C for 30 s, 70 °C for 30 s and 72 °C for 30 s, 3 cycles at 94 °C for 30 s, 68 °C for 30 s and 72 °C for 30 s, 4 cycles at 94 °C for 30 s, 66 °C for 30 s and 72 °C for 30 s, 4 cycles at 94 °C for 30 s, 64 °C for 30 s and 72 °C for 30 s, 36 cycles at 94 °C for 30 s, 62 °C for 30 s and 72 °C for 30 s, and a final step of 72 °C for 10 min. Another intergenic region of SL gene (SL-IR I and SL-IR II HN) was amplified with primers TCC, TC1 and TC2 to distinguish between TcI (350 bp) and TcII, TcV, TcVI (300 bp), and TcIII, TcIV (not amplified). Cycling conditions included 94 °C for 10 min, 5 cycles at 94 °C for 1 min, 67 °C for 1 min and 72 °C for 1 min, 5 cycles at 94 °C for 1 min, 65 °C for 1 min and 72 °C for 1 min, 5 cycles at 94 °C for 1 min, 63 °C for 1 min and 72 °C for 1 min, 30 cycles at 94 °C for 1 min, 61 °C for 1 min and 72 °C for 1 min, and a final step of 72 °C for 10 min. For D7 variable domain of 24Sα ribosomal subunit (24Sα rDNA HN), semi nested-PCR was used with primers D75 and D76 in the first round and D71 and D76 in the second round to differentiate between TcII, TcVI (140 bp), TcIII (125 bp), TcIV (140/145 bp) and TcV (125 or 125 + 140 bp). Cycling conditions consist in a first step at 94 °C for 10 min, 3 cycles at 94 °C for 30 s, 64 °C for 45 s and 72 °C for 1 min, 3 cycles at 94 °C for 30 s, 62 °C for 45 s and 72 °C for 1 min, 3 cycles at 94 °C for 30 s, 60 °C for 45 s and 72 °C for 1 min, 35 cycles at 94 °C for 30 s, 58 °C for 45 s and 72 °C for 1 min, and a final step of 72 °C for 10 min. The A10 fragment region (A10 HN) was amplified by semi-nested PCR with primers Pr1 and P6 in the first round. Cycling conditions were: the first step at 94 °C for 10 min, 35 cycles at 94 °C for 1 min and 65 °C for 1 min and 72 °C for 1 min, and a final step of 72 °C for 10 min.

Quantitative real-time PCR (second round) was performed using primers Pr3 and Pr1 to differentiate between TcII (Tm 80.2 °C) and TcVI (Tm 82 °C) (Fig. 1). Cycling conditions were the first step at 94 °C for 10 min, 35 cycles at 94 °C for 1 min and 60 °C for 1 min and 72 °C for 1 min, and a final step of 72 °C for 10 min.

The conventional PCR was set up in a volume of 30 μl containing 1× PCR buffer (pH 8.3), 250 μM dNTPs, 3 mM MgCl_2_, 5 U Platinum® *Taq* DNA polymerase, 1.67 μM of each primer, and 5 μl DNA solution. qPCR reactions were carried out in a final volume of 10 μl using 2× SYBR Green master mix buffer (Applied Biosystems), 0.5 μM of each primer, and 2 μl of DNA sample.

## Results

### DNA amplificability

**Fig. 2:**
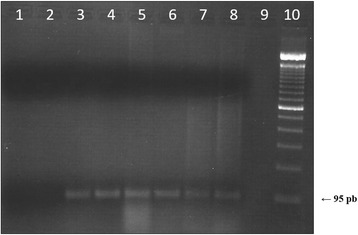
Amplification of the *rbcl* gene with primers VPRBCP1/VPRBCP2. Lane 1: sterile water (negative control), Lane 2: *Salmonella* sp. (negative control); Lane 3: rice; Lane 4: bean; Lane 5: soy; Lane 6: maize; Lane 7: acai juice; Lane 8: açai pulp; Lane 9: empty; Lane 10: 100 bp DNA ladder. The arrow indicates the PCR product size, 95 bp

The amplificability of DNA (i.e. monitoring the presence/absence of PCR inhibitors) extracted from all the samples was confirmed through the visualization of 95 bp amplicons resulting from the amplication of *rbcl* gene (Fig. 2). No PCR inhibition was observed following our methodology.

### Detection of *T. cruzi* DNA

**Fig. 3:**
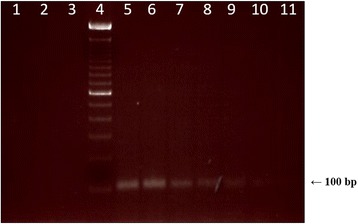
Limit of detection of *T. cruzi* in açai samples with primers Tc189F/Tc189R. *T. cruzi* DNA (from 75 pg to 2.4 pg) was mixed with 240 ng of açai DNA before the PCR assay using Tc189F/189R primers. Lane 1: sterile water (negative control); Lane 2: *T. rangeli* DNA (5 ng); Lane 3: Açai without *T. cruzi* (negative control); Lane 4: 100 bp DNA ladder. *T. cruzi* DNA: Lane 5: 75 pg; Lane 6: 45 pg; Lane 7: 24 pg; Lane 8: 15 pg; Lane 9: 9 pg; Lane 10: 3 pg; Lane 11: 2.4 pg

**Fig. 4:**
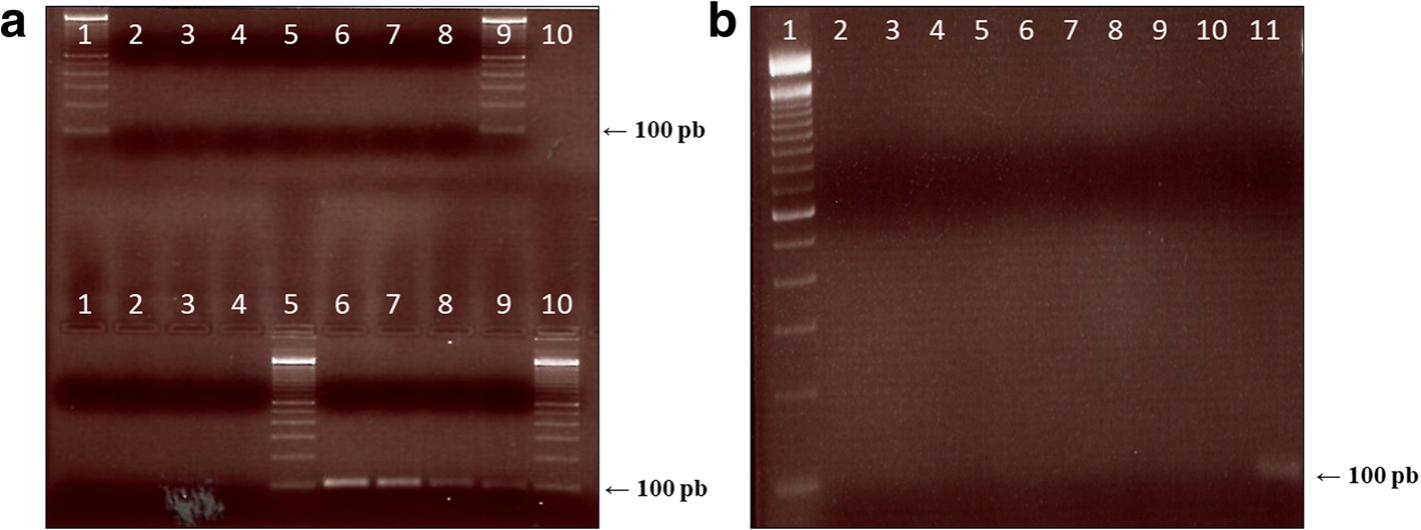
Analytical sensitivity and specificity of Tc189F/Tc189R primers to trypanosomatids and other microorganisms. PCR assays using primers Tc189F/Tc189R were performed with two ng DNA of trypanosomatids, yeast, fungi and bacteria. **a** Specificity assay (Non-detectable PCR). Upper lanes - Lane 1: 100 bp DNA ladder; Lane 2: sterile water (negative control); Lane 3: *T. rangeli*; Lane 4: *T. cervi*; Lane 5: *T. lewisi*; Lane 6: *T. mega*; Lane 7: *L. amazonensis*; Lane 8: *L. braziliensis*; Lane 9: 100 bp DNA ladder; Lane 10: no template. Bottom lanes - Lane 1: *L. guyanensis*; Lane 2: *L. lainsoni*; Lane 3: *L. naiffi*; Lane 4: *L. shawi*; Sensitivity assay (detectable PCR). Lane 5: 100 bp DNA ladder; Lane 6: *T. cruzi* Dm28c clone (TcI); Lane 7: *T. cruzi* Y strain (TcII); Lane 8: *T. cruzi* INPA 222 strain (TcIII); Lane 9: *T. cruzi* CL Brener clone (TcVI); Lane 10: 100 bp DNA ladder. **b** Specificity assay (continuation). Lane 1: 100 bp DNA ladder; Lane 2: *A. alternat*e; Lane 3: *B. cinerea*; Lane 4: *O. polymorpha*; Lane 5: *S. cerevisae*; Lane 6: *B. cereus*; Lane 7: *C. sakasaki*; Lane 8: *E. coli*; Lane 9: *Salmonella* sp.; Lane 10: *S. aureus*; Lane 11: *T. cruzi* CL Brener clone (TcVI)

Prior to the analysis of commercial açai-based samples, the limit of detection (LOD) and analytical sensitivity and specificity of PCR assays using primers Tc189F and Tc189R were evaluated. In Figs. 3, 2.4 ng of DNA extracted from non-contaminated açai was mixed with *T. cruzi* DNA, to create dilutions from 75 pg to 2.4 pg of parasite DNA. The 100 bp band, corresponding to the amplification of the telomeric junction of *T. cruzi* was observed from as little as 3 pg of *T. cruzi* DNA, which was considered LOD of the assay. In Fig. 4, the analytical sensitivity and specificity of PCR was evaluated using 2 ng of DNA from trypanosomatids, yeasts, fungi and bacteria. This assay successfully amplified DNA from four of the six *T. cruzi* DTUs (TcI, TcII, TcIII and TcVI) using primers Tc189F/Tc189R; DNA from all strains tested was amplified successfully. These primers did not amplify genomic DNA from other trypanosomatids (*T. rangeli*, *T. cervi*, *T. lewisi*, *T. mega*, *L. amazonensis*, *L. braziliensis*, *L. guyanensis*, *L. lainsoni*, *L. naiffi* and *L. shawi*), yeasts (*O. polymorpha* and *S. cerevisae*), fungi (*A. alternate* and *B. cinerea*), or bacteria (*B. cereus*, *C. sakasaki*, *E. coli*, *Salmonella* sp. and *S. aureus*).

**Fig. 5:**
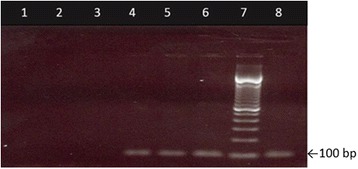
Amplification of *T. cruzi* DNA in açai samples with primers Tc189F/Tc189*r. *Lane 1: no template; Lane 2: sterile water; Lane 3: *T. rangeli* (negative control); Lanes 4–6: açai samples; Lane 7: 100 bp DNA ladder; Lane 8: *T. cruzi* (positive control)

Eleven of the 47 (23.4%) samples collected in 2010 were positive for *T. cruzi* DNA, as indicated by the 100 bp amplicon visualized via agarose gel electrophoresis (Fig. 5). From the samples collected in Rio de Janeiro, 8 (47%) of 17 samples were PCR-positive for *T. cruzi* DNA: açai juice (*n* = 2), açai with guaraná (*n* = 1), açai with guaraná and strawberry (*n* = 2), açai with guaraná and banana (*n* = 2) and açai with guaraná, strawberry and acerola (*n* = 1). Among the samples collected in Pará, 3 of 30 (10%) samples were PCR-positive: açai juice (*n* = 1) and açai fruit (*n* = 2), all collected in the “Feira do açai”, at the Belém city (Table 1). Comparative analysis of PCR positivity showed no statistically significant difference (difference between independent proportions test, *P* = 0.5722) between the two states.

**Table 1:** Detection of *T. cruzi* DNA in samples collected in Rio de Janeiro and Pará States

Sample type	Collection place	Collection year	No. of positive samples/total no. of samples
Açai juice	RJ	2010	2/9
	PA	2010	1/9
	PA	2015	0/4
Açai pulp	PA	2010	0/2
	PA	2012	1/15
	PA	2013	1/6
Açai with guaraná or fruits	RJ	2010	6/8
Açai candy	PA	2010	0/1
Chocolate bonbon with açai	PA	2010	0/1
Açai ice cream	PA	2010	0/1
Açai popsicle	PA	2010	0/1
Açai with rice porridges	PA	2010	0/2
Açai seed	PA	2010	0/2
Açai fruit	PA	2010	2/11
	PA	2012	1/15
	PA	2013	0/5

*Abbreviations*: *PA* Pará, *RJ* Rio de Janeiro

From the 93 samples collected in 2011, 2012 and 2015 in Pará and Rio de Janeiro states, only three samples were positive. From the 44 samples collected in Pará, two samples collected in 2012 were positive: one açai pulp and one açai fruit. In the açai pulp sample, *T. cruzi* DNA was also detected. None of the 48 samples collected in Rio de Janeiro between 2011 and 2012 were positive for parasite DNA by PCR (Table 1).

### Detection of triatomine DNA

**Fig. 6:**
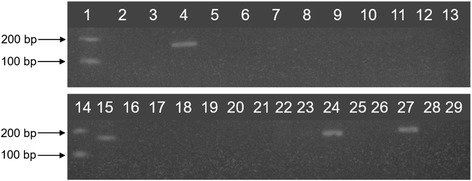
Amplification of triatomine DNA in açai samples with primers P2B/P6*r.* Lanes 1 and 14: 100 bp DNA ladder; Lanes 2 and 3: no template; Lane 4: uninfected triatomine (positive control); Lanes 5–7 and 9–10: açai with fruit; Lane 8: açai with guaraná; Lanes 11–13: açai; Lanes 15 and 16: açai fruit; Lanes 17 and 18: açai; Lane 19: *T. cruzi*; Lane 20: *T. rangeli*; Lane 21: açai pulp; Lane 22: açai fruit; Lane 23: *T. cruzi*; Lane 24: Triatomine spiked with *T. cruzi* (positive control); Lanes 25 and 26: *T. cruzi*; Lane 27: uninfected triatomine (positive control); Lane 28: *T. rangeli*, Lane 29: *T. cruzi*

The samples positive for *T. cruzi* DNA were analyzed for triatomine DNA by conventional PCR, to verify the simultaneous presence of parasite and invertebrate vector DNA in the same sample. From 13 samples analyzed, only 1 sample of açai juice showed an amplicon of 163 bp (Fig. 6), indicating the presence of triatomine DNA. This sample was collected in the Pará state, in 2010.

### *Trypanosoma cruzi* genotyping

**Fig. 7:**
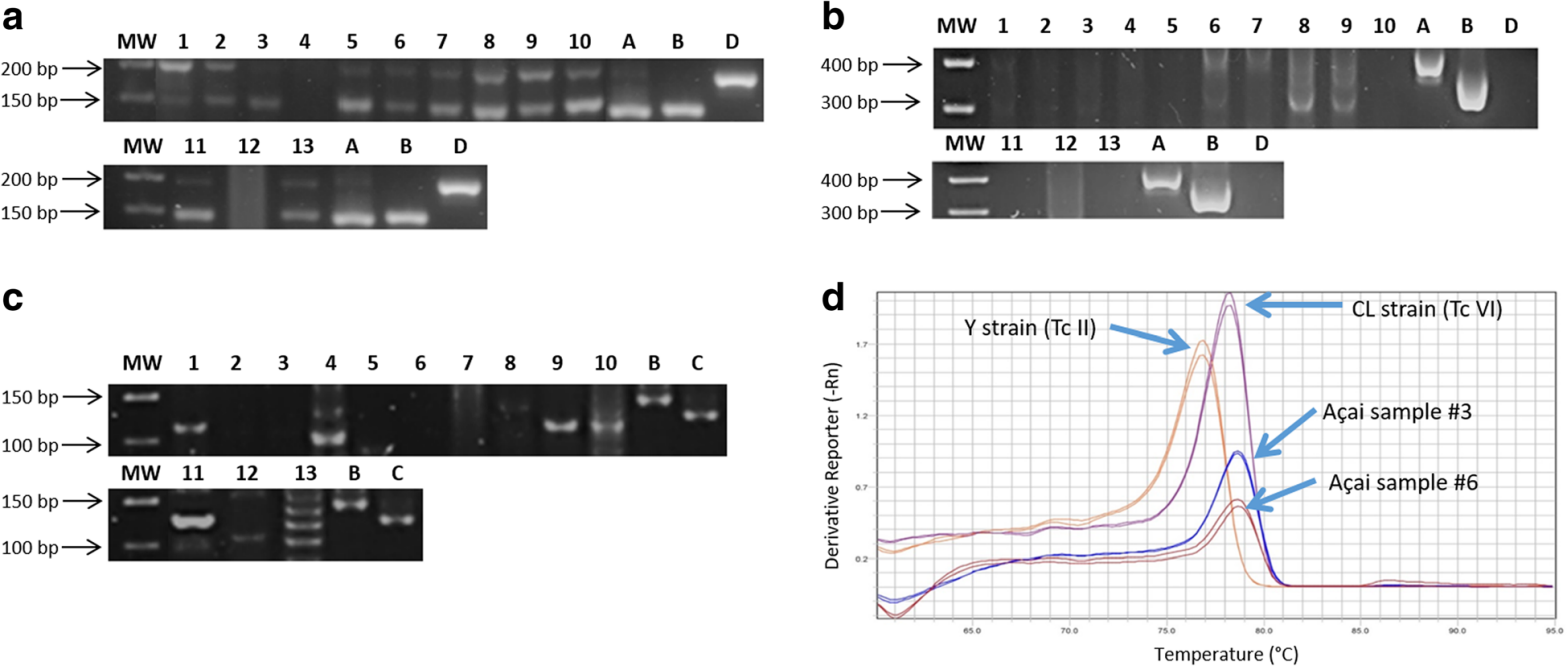
Amplified fragments generated by SL-IRac (**a**), SL-IR-I and II (**b**), and 24S rDNA (**c**) targets and melting curves generated by the amplification of nuclear fragment A10 in positive controls (**d**). **a**-**c** Lane MW: 50 and 100-bp DNA ladder; Lanes 1–13: açai samples; Lane A: Dm28c strain (Reference TCI); Lane B: Y strain (Reference TcII); Lane C: INPA 222 strain (Reference TcIII); Lane D: D INPA 4167 strain (Reference TcIV). **d** Melting curves for different amplification products indicated by arrows on the graph: Y strain (Reference TcII), CL Brener strain (Reference TcVI), Açai 3 sample, Açai 6 sample

From the samples positive for *T. cruzi* DNA, 13 were submitted for genotyping using SL intergenic region (SL-IR ac and SL-IR I and II), D7 variable domain of 24Sα ribosomal subunit, and A10 fragment region as targets. Figure 7 shows the amplified fragments generated by SL-IRac (a), SL-IR-I and II (b), and 24Sα rDNA (c). To differentiate TcII from TcVI, the melting curves generated by qPCR, corresponding to amplification of nuclear fragment A10 are also shown (Fig. 7d).

As shown in Table 2, food products containing açai showed a mixture of *T. cruzi* DTUs, with the prevalence of TcIII, TcV, and TcI, indicating different genotypes circulate in the same region, but with the predominance of DTUs associated with the sylvatic cycle of the parasite*.* All the analyzed samples represented mixed infections, but the identification of at least one DTU was possible in 10 samples (76.9%). From these, 5 (38.5%) were infected with TcIII, 5 (38.5%) with TcV, 4 (31%) with TcI, 2 (15.4%) with TcIV and one sample presented TcVI genotype. In four samples (30.8%), inconclusive results were obtained: two samples were also infected with TcII or TcVI (samples 8 and 11) and another two contained TcIII or TcIV genotypes (samples 6 and 7). Three samples, consisting of açai with guaraná, açai with guaraná and banana and açai with guaraná, strawberry and acerola, could not be genotyped (Table 2).

**Table 2:** Genotyping profile (DTU) of *T. cruzi* DNA isolated from açai samples

Sample	Sample code	Collection place	SL-IRac (bp)	SL-IR I e II (bp)	24S-α (bp)	A10 (°C)	DTU
1	AGF (S)	RJ	150 + 157 + 200	300 + 350	125	–	I + III + V
2	AGF (B)	RJ	157 + 200	300	125	–	III + V
3	AGF (B)	RJ	200	na	–	78.2	ni
4	AG	RJ	na	na	na	–	ni
5	AGF (S and A)	RJ	150 + 157 + 200	na	na	–	ni
6	AGF (S)	RJ	150 + 157 + 200	300 + 350	na	78.2	I + VI+ (III or IV)
7	A	RJ	150 + 200	350	na	–	I+ (III or IV)
8	A	RJ	157 + 200	300	140	na	IV+ (II or VI)
9	A	RJ	150 + 157 + 200	300 + 350	125	–	I + III + V
10	F	PA	157 + 200	na	125	–	III + V
11	F	PA	157 + 200	na	140	na	IV+ (II or VI)
12	P	PA	na	300	na	–	ni
13	F	PA	157 + 200	na	125	–	III + V

*Abbreviations*: *A* açai, *P* pulp, *F* fruit, *AG* açai with guaraná, *AGF* açai with guaraná and fruit, *S* strawberry, *B* banana, *S* and *A* strawberry and acerola, *RJ* collected in Rio de Janeiro, *PA* collected in Pará, *na* no amplification, *ni* not identified, not performed, *DTU* discrete typing unit

## Discussion

*Trypanosoma cruzi* and *T. rangeli* are protozoan species of sympatric occurrence between South and Central America. They share the same invertebrate hosts as well as wild and domestic mammalian hosts including humans [15]. Considering that the contamination of açai fruits occurs by faeces deposition or triatomine maceration during the açai fruit processing, the PCR-based method used here can differentiate the two species and is highly recommended to avoid false-positive results. The conventional PCR with primers described by Chiurillo et al. [12] resulted in a robust method to detect *T. cruzi* without non-specific amplification of *T. rangeli* DNA in the açai-based matrix. In fact, the analytical specificity assay indicates 100% specificity to *T.cruzi* detection. The selectivity of this set of primers could be explained by the high interspecific variability of the trypanosomatids sub-telomeric sequences used as targets. Thus, the primers Tc189F and Tc189R were used instead of primers that anneal to kinetoplast minicircle DNA (kDNA) and nuclear satellite DNA (satDNA) as those sets of primers may amplify DNA from *T. rangeli* [16, 17].

Besides the considerable but not statistically significant in *T. cruzi* DNA detection between samples from Pará and Rio de Janeiro states, the differences in positivity among the type of food were quite remarkable. In the Rio de Janeiro state, the consumption of açai pulp with guaraná syrup is common, because açai is not part of local diet. Curiously, the majority of samples positive for *T. cruzi* DNA consisted of guaraná syrup and fruits such as strawberry, banana and acerola. These samples were produced by food industries that should be applying strict manufacturing practices, including raw material selection, to assure food safety for consumption.

Previous studies have shown that heating above 45 °C and pasteurization can inactivate *T. cruzi* in açai pulp [18, 19]. In contrast, there is no consensus about the efficacy of freezing to kill the parasite in pulp. Neves et al. [20] demonstrated that *T. cruzi* is inactivated after 2 h at -20 °C. However, Barbosa-Labello et al. [21] showed that *T. cruzi* maintained its virulence after staying in contact with frozen pulp for up to 26 h. In addition, carbohydrates in guaraná syrup could act as cryopreservant to maintain parasite viability. Although some rules have been established for açai manufacture and processing in Brazil [22, 23], the methods still require validation for quality control of commercialized products. Thus, there is a possibility of açai-based foods enhancing the risk of oral Chagas disease transmission. However, more studies using animal models are needed to properly evaluate the infection rate of viable *T. cruzi* from açai-based products.

Triatomines are numerous, diversified, and colonize temperate, tropical, and subtropical ecotypes on the American continent. *Rhodnius* spp. and *Panstrongylus megistus* are highly associated with palm trees and bromeliads [24, 25]. Curiously, many kinds of tropical fruits such as palm tree fruits show a soft pericarp similar to açai fruit, which is associated with Chagas disease transmission in Brazil [26]. Diaz-Albiter et al. [26] described the first report of phytophagy and sugar feeding of *Rhodnius prolixus*, suggesting that plants may have an important nutritional role in triatomine maintenance and could contribute to increasing its life-time. These authors also verified that all five *Rhodnius* instars, always considered strict hematophagous, can feed themselves with 10% glycose solution and can get nutrients through the perforation of vegetal tissues such as tomato. Therefore, the acquisition of nutrients from flowers and fruits can be an additional explanation for the association of triatomine with tropical plants.

It is known that when triatomines are starved for a long period there is a population decrease of *T. cruzi* in the rectum and an increase of infecting metacyclic trypomastigote forms [27, 28]. Passos et al. [29] analyzed mice inoculated with *T. cruzi* after contact with açai and showed that a longer period of contact between parasite and açai pulp enhances the virulence and anticipates the onset of parasitemia and death. Xavier et al. [30] demonstrated that the urban cases of ACD were related to wild infected triatomines that were accidentally transported in boats from the islands to açai planting areas. In our work, we analyzed a sample of açai gathered from an urban area “Feira de Açai” that showed a PCR positive reaction for the presence of triatomine DNA. This demonstrated a tenuous link between triatomines and açai and may suggest these insects are the source of food contamination with *T. cruzi*. This finding highlights the need for research on the phytophagia of triatomines, the effect of açai in development, reproduction, and survival of these insects, and the behaviour of *T. cruzi* population in triatomines when the diet is based on phytophagia and especially açai. This anomalous finding highlights the need for an eco-epidemiological study to assess the frequency of contamination inside palm trees.

Fregonesi et al. [31] analyzed 30 frozen açai samples (pulp and juice) gathered from markets and snacks bars in Ribeirão Preto, SP and verified that 63% did not meet the standards set by local authorities, i.e. total solids vs moisture content. In addition to moisture content, this study found 50% of edible products were composed of insect fragments, mites, sand crystals, and human hair, which demonstrated quality control failures in food production practices. Another sample presented a rodent pelage, being considered unfit for human consumption according to Brazilian legislation [32]. Freitas et al. [33] also found samples presenting insect fragments. Fragments of insects found in some foods are not commonly identified because of their small size. Even with the initial data reported here, we can raise the possibility that the presence of tiny fragments in açai samples can be related to contamination with insect vectors that were infected with *T. cruzi* making the product a vehicle of ACD by oral transmission.

The lack of uniformity in the production of açai pulp and açai-based products combined with inadequate sanitary quality may lead to the devaluation of the food product. Given the variety of new açai-based products being launched in Brazil, the development of analytical tools becomes a challenge for laboratories testing complex food matrices. Samples collected from Pará in 2012 were part of a monitoring program led by local regulatory health authorities to establish sanitary procedures for manipulation and marketing of açai juice throughout the production chain. After that, 30 samples (15 açai fruits and 15 açai pulps) were gathered from 15 different commercial establishments. Only one açai pulp sample was positive for *T. cruzi* DNA demonstrating that there were still gaps in the implementation of good manufactures practices despite these efforts. In 2015, this scenario changed with no sample positive for *T. cruzi* DNA. Taken together, our results emphasize the presence of parasite DNA as a marker of the absence of good manufacturing practices in acai-based commercial food. It is important to point it out that the detection of *T. cruzi* DNA cannot be directly associated with the oral transmission of Chagas disease by the consumption of açai as DNA can also be detected from recently inactivated parasites. Nevertheless, previous studies showed that, at high humidity, *T. cruzi* in triatomine faeces preserves their mobility and infectivity up to 30 min at 33 °C [34]. Furthermore, *T. cruzi* can remain infective inside dead triatomines stored at 10 °C for six days and between 26 and 30 °C for at least two months [35]. In açai fruit, *T. cruzi* can be viable, at room temperature, for up to nine hours after contamination [20] and in açai pulp for up to 28 h after contamination [36]. Therefore, a robust study using a molecular marker specific to *T. cruzi* viability in açai-based food, such as the parasite RNA, remains necessary to investigate the potential of infected açai as a source of contamination. The *T. cruzi* genotyping analysis here, demonstrated that açai-based products contaminated with *T. cruzi* DNA showed a mixture of DTUs, including TcI, TcIII and TcIV. These genotypes are in agreement with other studies in the literature [4, 5], which showed a correlation between triatomines and *Didelphis* in food outbreaks.

The genotype TcI seems to be prevalent in the Amazon Basin [37] and is associated with ACD outbreaks by oral transmission [38]. The sylvatic vectors usually include species of *Rhodnius*, *Panstrongylus*, *Triatoma* and *Eratyrus.* The sylvatic hosts are usually arboreal and semi-arboreal animals, especially *Didelphis*, arboreal rodents, primates, *Tamandua*, and terrestrial rodents. The main ecotypes are palm, tree holes, rocky landscapes, and terrestrial Amazonian environments.

The genotype TcIII shows ecological terrestrial and fossorial niches, and armadillos, especially species of *Dasypus*, *Chaetophractus*, *Euphractus* and *Didelphis* and *Monodelphis* are the sylvatic hosts [4]. *Panstrongylus geniculatus* are the sylvatic vectors, widespread in South America. This genotype is rare in humans with few cases described in Amazon and Brazilian south-east region [39]. It is also rare in domestic dogs with a few reports in Mato Grosso do Sul [40]. The acute cases attributed to this DTU occurred in the Brazilian Amazon, showing subclinical symptoms.

The TcV genotype is found in the Southern Cone (Brazil, Paraguay, Uruguay, Argentina and Chile), in the extreme South of Brazil, and great Gran Chaco region (Bolivia, Argentina, Paraguay and Brazil). Patients from the Southern cone show cardiac forms and present megasyndromes as symptoms of Chagas disease [4]. Although the description of TcV in ecological niches, sylvatic vectors, and hosts (the most usual are armadillos, especially *Dasypus* spp. and *Euphractus* spp., and rodents *Octodon* spp.) is rare, Araujo et al. [41] have made the first report of TcV infecting a wild host, the caviomorph rodent species *Thrichomys laurentius* in Brazil. In addition, Lima et al. [42], showed, for the first time, the presence of hybrid DTUs (Tc V or Tc VI) from the triatomine *Rhodinus pictipes* and a dog in the state of Pará in the Brazilian Amazon. Here, five (38.5%) of the açai samples presented the genotype TcV, mixed with other DTUs. Even more unusual, the prevalence of TcV is a consequence of the epidemiological changes occurring in the Amazon Basin area.

Valente et al. [43] studied an outbreak of ACD by açai consumption in Mazagão (Amapá) with 96 affected subjects. They identified 68 triatomines belonging to *Rhodnius pictipes* (*n* = 66) and *Panstrongylus geniculatus* (*n* = 2) captured in the local area. Of these, 45 (43 *Rhodnius pictipes* and 2 *Panstrongylus geniculatus*) were infected with *T. cruzi.* Thirteen isolates (eight from human and five from *Rhodnius pictipes)* showed Z3 and TcI genotypes, using the *T. cruzi* mini-exon as a target for genotyping. Also, a mixed infection of Z3 and *T. rangeli* in two isolates of *Rhodnius pictipes* was observed*.* This method does not distinguish between TcIIa from TcIIc and classifies both genotypes as Z3. The authors assumed that these isolates belong to genotype TcIIa that has been associated with human disease. Therefore, following the classification of Zingales et al. [44], these isolates correspond to DTU TcI and TcIV (former TcIIa).

Steindel et al. [45] genotyped 13 *T. cruzi* isolates from humans, opossums (*Didelphis aurita* and *Didelphis albiventris*), and the vector (*Triatoma tibiamaculata*) involved in an outbreak of ACD in Santa Catarina in 2005, by ingestion of sugar cane juice. The isolates were characterized by multilocus enzyme electrophoresis (MLEE) and analysis of the spliced-leader and 24Sα rDNA genes. The molecular genotyping demonstrated that all isolates from humans belong to TcII, while the isolates from opossum belong to TcI and the isolates from triatomines showed a mix contamination with TcI and TcII, according to the classification (in Anonymous [46]).

Brazil is still in the early stages of controlling Chagas disease transmission by açai-based products, despite important recent prevention strategies [6]. Recently, quantitative real-time PCR to detect and quantify *T. cruzi* from açai-based samples with high sensitivity have been developed by Mattos et al. [47] and Souza Godoi et al. [48], reaching LOD of 0.1 parasite equivalent (0.35 fg/μl) and 0.44 parasite equivalents (4–10 ng/μl). Nevertheless, it is still necessary to develop more scientific researcher related to the viability of the parasite in different types of food, the mechanisms of food infection, food preservation technologies, methodologies of *T. cruzi* detection in food, and other strategies to upgrade the understanding of oral transmission and advance its epidemiological features, prevention, and control [49]. It also remains necessary to develop more strategies to assure the safety of açai products while keeping the nutritional and sensorial properties. Thus, the implementation of good hygienic practices, good manufacturing practices, and joint work between scientists and açai producers are essential to improve product quality.

## Conclusions

This work showed, to our knowledge for the first time, a sanitary assessment of manufactured açai-based products through the detection of *T. cruzi* DNA. The samples were produced in all Brazilian regions and commercialized in Rio de Janeiro and Pará states. The PCR used in this study could detect small quantities of *T. cruzi* DNA in an açai-based matrix. Also, the genotyping analysis demonstrated that açai-based products contaminated with *T. cruzi* DNA showed a mixed contamination with the predominace of DTUs TcI, TcIII, and TcIV.

## Abbreviations

ACD: acute Chagas disease
ChD: Chagas disease
CMRVS: Coleção de Microorganismos de Referência of the National Quality Control Institute (Rio de Janeiro, Brazil)
COLPROT: Coleção de Protozoários da Fundação Oswaldo Cruz, Rio de Janeiro, Brazil
DTU: discrete typing unit
Fiocruz: Oswaldo Cruz Foundation
HN: heminested
INCQS: National Institute of Quality Control in Health, Oswaldo Cruz Foundation
IOC: Oswaldo Cruz Institute, Oswaldo Cruz Foundation
IR: intergenic region
LACEN /PA: Central laboratory of Public Health of Pará
MLEE: multilocus enzyme electrophoresis
SES: State Secretary of Health of Rio de Janeiro
SESPA: State Secretary of Health of Pará
SL: spliced leader
TcI, TcII, TcIII, TcIV, TcV and TcVI: *Trypanosoma cruzi* discrete typing units I, II, III, IV, V and VI
Tm: melting temperature
